# Mechanical regulation of LINC complex function: structural basis, physiological roles, and disease mechanisms in transnuclear mechanotransduction

**DOI:** 10.3389/fcell.2026.1946704

**Published:** 2026-09-30

**Authors:** Shuoshuo Shao, Zhu Zhu, Huaiquan Liu

**Affiliations:** 1 Guizhou University of Traditional Chinese Medicine, Guiyang, Guizhou, China; 2 Guiyang Healthcare Vocational University, Guiyang, Guizhou, China

**Keywords:** chromatin regulation, LINC complex, mechanotransduction, nuclear mechanics, nucleus-cytoskeleton coupling, SUN-KASH linkage

## Abstract

How mechanical stimuli cross the nuclear envelope and are converted into nuclear structural and transcriptional responses is a central question in understanding cellular mechanical adaptation and its failure. The LINC complex is composed of inner nuclear membrane SUN proteins and outer nuclear membrane KASH/Nesprin proteins, and establishes a continuous transnuclear mechanical pathway through its connections with the nuclear lamina and the cytoskeleton. Current evidence indicates that the LINC complex is not a passive molecular bridge. Instead, through SUN-KASH pairing, Nesprin isoforms and perinuclear cytoskeletal linkages, it selects and distributes mechanical inputs from different sources, thereby regulating nuclear positioning, nuclear prestress, nuclear pore transport, chromatin organization and mechanosensitive transcriptional programs. These mechanisms participate in development and in the mechanical adaptation of skeletal and cardiac muscle. When disrupted, they may manifest as global mechanical uncoupling, local stress overload and imbalanced nuclear responses, and are associated with pathological processes including Emery-Dreifuss muscular dystrophy, dilated cardiomyopathy, neurological disease, premature ageing and tumour invasion. On the basis of this evidence, we propose a continuous framework of “mechanical input selection, transmembrane force distribution, nuclear response conversion and tissue outcome” to explain tissue selectivity and phenotypic heterogeneity in LINC-related diseases, and to inform patient variant assessment, disease stratification and precise intervention in aberrant force-transmission pathways.

## 1 Introduction

Cells reside in complex and dynamic mechanical microenvironments. They sense a range of mechanical cues, including extracellular matrix stiffness, stretch, compression, shear stress and cell-cell interactions, and convert these cues into biological signals that regulate cell shape maintenance, migration, proliferation, differentiation and fate decisions (Di et al., 2023; Ke et al., 2025; Xie et al., 2023). This process, known as mechanotransduction, converts mechanical stimulation into intracellular biochemical reactions and transcriptional programs, and is essential for adaptation to the tissue microenvironment, maintenance of structural homeostasis and regulation of organ function (Cao et al., 2024; Wagh et al., 2021). In mechanically sensitive tissues such as skeletal muscle, myocardium, blood vessels and the nervous system, stable mechanotransduction is particularly important for tissue development, functional maintenance and injury repair (Jin et al., 2024; Zhao et al., 2026; Zhao et al., 2025; Zimkowska et al., 2026). The nucleus is not only the repository of genetic material and a central regulator of gene expression, but also an important mechanosensitive organelle. After external mechanical forces are transmitted to the nucleus through the cytoskeleton, they can affect nuclear morphology, nuclear positioning, nuclear stiffness, nuclear envelope tension and nuclear pore complex function, and can further alter spatial chromatin organization, nuclear translocation of transcription factors and mechanosensitive gene expression (Rajan et al., 2026; Yang et al., 2025; Zhan et al., 2026). Thus, mechanotransduction is not confined to cytoplasmic signalling cascades. Its ultimate effects converge on nuclear structural remodelling and transcriptional reprogramming. Clarifying how mechanical forces are transmitted from the extracellular environment to the nucleus and converted into stable biological responses within the nucleus is therefore one of the central scientific questions in current mechanobiology.

The LINC (linker of nucleoskeleton and cytoskeleton) complex provides a key structural basis for understanding how mechanical forces enter the nucleus. This complex is composed of inner nuclear membrane SUN proteins, outer nuclear membrane KASH/Nesprin proteins, and associated components linked to the nuclear lamina and cytoskeleton. Together, these elements form a force-bearing axis that spans the nuclear envelope and connects the cytoskeleton with the nucleoskeleton (Jahed et al., 2021; Khilan et al., 2021). On the cytoplasmic side, KASH/Nesprin proteins connect with cytoskeletal networks such as actin, intermediate filaments and microtubules. On the nuclear side, SUN proteins interact with lamin A/C, emerin and chromatin-associated structures, allowing extracellular and cytoplasmic mechanical stimuli to cross the nuclear envelope and reach the nucleus. These inputs can then regulate nuclear envelope stability, nucleoskeletal assembly, chromatin conformation and gene transcription (King, 2023; Lityagina and Dobreva, 2021). Accordingly, the LINC complex is not only an important structural bridge that maintains nuclear positioning and nuclear morphology, but also a transnuclear mechanotransduction hub that couples mechanical stimuli to nuclear responses. Its structural integrity and dynamic regulation are therefore crucial for nuclear mechanical homeostasis. When the LINC complex or its lamina-associated components become dysfunctional, mechanical coupling between the cytoskeleton and nucleoskeleton may be weakened, leading to reduced nuclear structural stability, disordered mechanosensitive signalling and imbalanced gene expression (Bougaran and Bautch, 2024; Shen et al., 2026; Takata and Matsumura, 2022). In tissues such as skeletal muscle and myocardium, which are chronically exposed to cyclic contraction and stretch, such mechanical defects are more likely to be converted into nuclear damage and tissue functional decompensation, and contribute to diseases such as Emery-Dreifuss muscular dystrophy and dilated cardiomyopathy (Amiad Pavlov et al., 2025; Jabre et al., 2025; Zi-Yi et al., 2024). LINC-related abnormalities in nuclear mechanics are also associated with neurological disease, progeroid syndromes, and tumour cell migration and invasion (van Ingen and Kirby, 2021). These disease phenotypes suggest that the consequences of LINC complex dysfunction are not limited to nuclear positioning or nuclear envelope injury. Instead, they may involve a continuum of altered force transmission, chromatin organization and gene-expression regulation. Although studies in structural biology, cellular mechanics, nuclear transcriptional regulation and disease genetics have revealed important roles of the LINC complex, a sufficiently integrated cross-scale mechanistic explanation remains lacking. In particular, it remains unclear how mechanical stimuli are transmitted, amplified and converted into nuclear responses along the continuous cytoskeleton-SUN-KASH-lamina structural axis, and how these nuclear changes further shape tissue-specific function and disease susceptibility. By focusing on the continuous process of mechanical input selection, transmembrane force distribution, nuclear response conversion, and tissue adaptation or disease output, the structural basis, transmembrane force transmission, nuclear effects and disease mechanisms of the LINC complex can be placed within a unified cross-scale framework. Rather than treating these processes as separate structural, mechanical and pathological phenomena, this framework emphasizes the continuity across these levels: the assembly of the LINC complex and its coupling to distinct cytoskeletal systems determine how mechanical inputs are selected and distributed, and these differences in turn shape nuclear mechanical and transcriptional responses and ultimately manifest as tissue adaptation or disease phenotypes. This review was informed by searches of PubMed, Web of Science and Scopus for relevant literature published from database inception to July 2026, using combinations of terms including “LINC complex,” “SUN,” “KASH,” “Nesprin,” “mechanotransduction,” and “nuclear mechanics.” Priority was given to representative primary studies directly relevant to LINC structure, force transmission, nuclear responses and disease mechanisms, supplemented by recent reviews. The proposed integrative framework is summarized in Figure 1.

**FIGURE 1:**
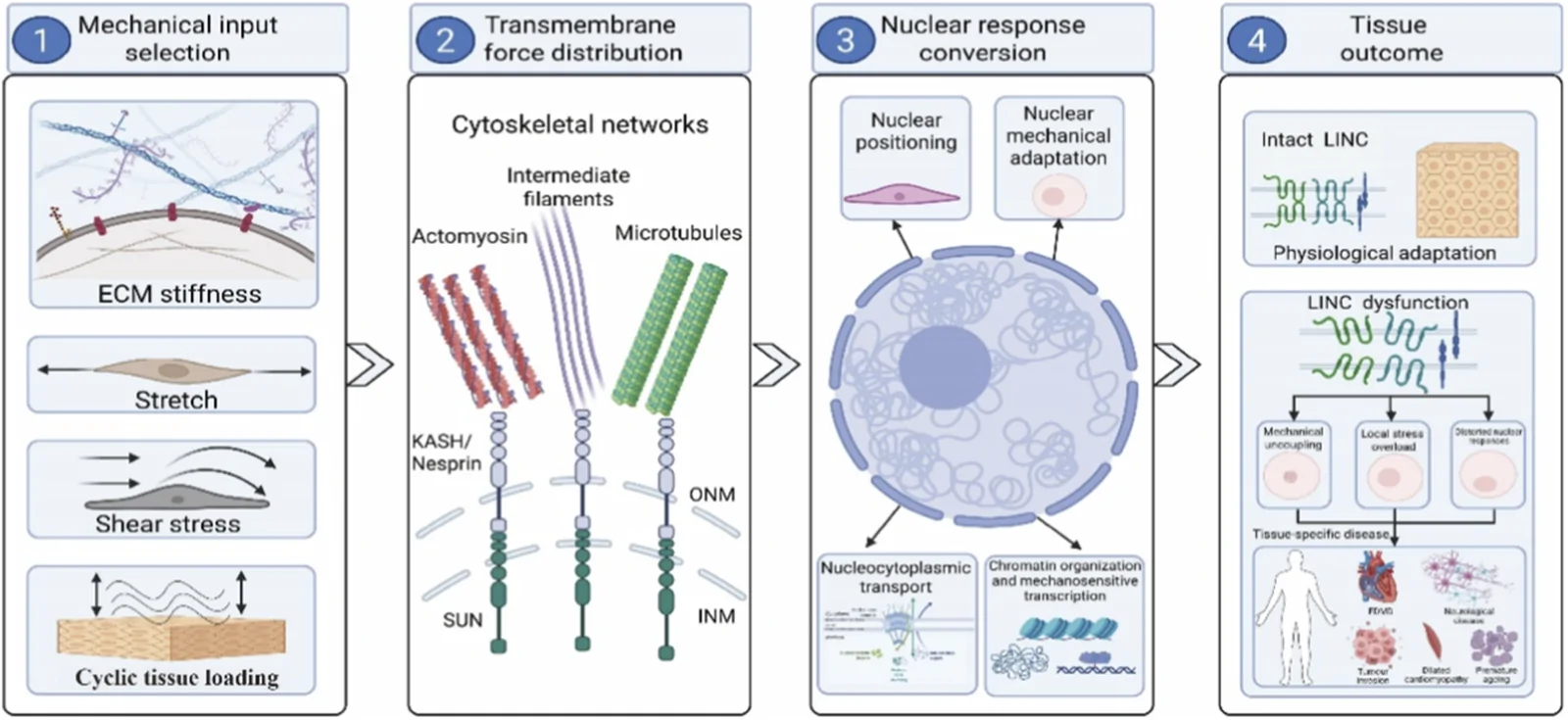
An integrative framework for LINC-mediated transnuclear mechanotransduction. Mechanical inputs are selected and transmitted through cytoskeletal networks and the LINC complex, producing nuclear mechanical, transport and chromatin responses. An intact LINC axis supports physiological adaptation, whereas LINC dysfunction may produce mechanical uncoupling, local stress overload and distorted nuclear responses, ultimately contributing to tissue-specific disease. Abbreviations: EDMD, Emery–Dreifuss muscular dystrophy; INM, inner nuclear membrane; ONM, outer nuclear membrane.

## 2 Structural composition of the LINC complex and its role as a transnuclear mechanical bridge

### 2.1 The core SUN-KASH linkage and transnuclear architecture

The LINC complex is a molecular connection device that spans the nuclear envelope. Its core function is to connect the cytoplasmic cytoskeleton with the nuclear nucleoskeleton, thereby establishing mechanical continuity between the cytoplasm and the nucleus (Kirby and Lammerding, 2018). A canonical LINC complex is mainly composed of SUN-domain proteins located in the inner nuclear membrane and KASH/Nesprin proteins located in the outer nuclear membrane (Kuwako and Suzuki, 2024). The C-terminal domain of SUN proteins extends into the perinuclear space, whereas the C-terminal KASH domain of KASH/Nesprin proteins crosses the outer nuclear membrane and enters the perinuclear space. These two domains bind specifically within the perinuclear space, forming the core SUN-KASH connection unit that links the inner and outer nuclear membranes (Gurusaran and Davies, 2021; Jahed et al., 2021; Sosa et al., 2012). Spatially, the SUN-KASH connection gives the LINC complex the basic capacity to bear force across the nuclear envelope. SUN proteins usually form oligomeric structures through their luminal SUN domains and bind KASH domains to create a relatively stable transmembrane connection interface. This interface, located in the perinuclear space between the inner and outer nuclear membranes, not only maintains the localization of the LINC complex at the nuclear envelope, but also provides the structural basis for mechanical force generated by the cytoskeleton to cross the nuclear envelope and enter the nucleus (McGillivary et al., 2023; Zhan et al., 2026). At the structural level, SUN proteins form a trimeric platform that binds three KASH peptides at the interfaces between adjacent SUN protomers. The C-terminal PPPT motif of canonical KASH1/2 peptides fits into a hydrophobic pocket at the SUN trimer interface, while the KASH-lid of the SUN domain and a conserved SUN-KASH disulfide bond further stabilize the interaction (Sosa et al., 2012; Wang et al., 2012). Although SUN1 and SUN2 share the conserved SUN-domain architecture, SUN-KASH recognition is not uniform across different KASH partners. High-resolution SUN2 structures with KASH peptides from Nesprin-3, Nesprin-4, and KASH5 showed similar recognition of the C-terminal residues but distinct conformations of the extended KASH regions, revealing KASH-specific structural variation beyond the conserved C-terminal recognition mode (Cruz et al., 2020). More recent structural and biochemical studies of SUN1- and SUN2-containing complexes have provided evidence for head-to-head 6:6 SUN-KASH assemblies and have suggested differences in complex stability and higher-order organization (Gurusaran and Davies, 2021). Thus, the SUN-KASH linkage is not merely a protein-protein interaction interface. It is the core structural unit through which the LINC complex achieves transnuclear mechanical coupling. This basic architecture gives the LINC complex clear directionality and functional division. The cytoplasmic ends of KASH/Nesprin proteins connect with actin, intermediate filaments or microtubule networks and receive mechanical inputs from the cytoplasmic side. The nuclear ends of SUN proteins interact with the nuclear lamina and chromatin-associated structures, allowing mechanical forces to be transmitted further into the nucleus (Liu et al., 2026; Zi-Yi et al., 2024). In this way, the LINC complex forms a continuous mechanical axis composed of cytoskeleton, KASH/Nesprin proteins, SUN proteins and the nuclear lamina, providing the basic structural framework for subsequent transnuclear mechanical signalling, nuclear envelope responses and nuclear transcriptional regulation.

### 2.2 Cytoskeletal coupling mediated by KASH/Nesprin proteins

KASH/Nesprin proteins are located in the outer nuclear membrane and serve as core effector elements through which the LINC complex connects to cytoskeletal networks. Their C-terminal KASH domains bind inner nuclear membrane SUN proteins within the perinuclear space, forming transnuclear SUN-KASH molecular bridges that support the transmission of cytoskeleton-derived mechanical forces to the nuclear lamina and intranuclear structures (Lombardi et al., 2011; Sosa et al., 2012). Unlike SUN proteins, which mainly provide transnuclear anchoring, the cytoplasmic domains of Nesprin proteins determine which cytoskeletal inputs can be received by the LINC complex. Nesprins are therefore central to mechanical input selection and routing. Different members of the Nesprin family couple specifically to distinct cytoskeletal systems through sequence differences in their cytoplasmic binding domains. Nesprin-1 and Nesprin-2 giant contain N-terminal actin-binding domains that directly bind F-actin, thereby transmitting tensile forces generated by the actin network, migration-associated tension and signals from stress-fibre rearrangement to the nucleus (Davidson and Cadot, 2021; Leong et al., 2023). In polarized migrating cells, Nesprin-2G and SUN2 assemble into TAN lines arranged along dorsal actin bundles. These transmembrane actin-associated nuclear lines couple retrograde actin flow to the nuclear envelope and thereby drive nuclear positioning and centrosome reorientation, indicating that Nesprin-2-mediated actin-LINC coupling has a direct force-transmission function (Luxton et al., 2010).

Beyond the actin network, Nesprin-3 anchors the outer nuclear membrane to the intermediate filament system through the adaptor protein plectin. Wilhelmsen et al. showed that Nesprin-3 lacks a typical actin-binding domain and instead couples through plectin to intermediate filament networks such as keratin, providing molecular evidence that the LINC complex can receive mechanical input from intermediate filaments (Wilhelmsen et al., 2005). In vascular endothelial cells, Nesprin-3 also regulates perinuclear cytoskeletal organization, maintenance of cell morphology and flow-induced cell polarization, suggesting a tissue- and cell-type-specific mechanical regulatory role under mechanical load (Morgan et al., 2011). Microtubule-associated mechanical inputs are mainly mediated by KASH proteins such as Nesprin-4 and KASH5. Nesprin-4 is an outer nuclear membrane protein that binds the motor protein kinesin-1. Its nuclear envelope localization depends on interaction with SUN proteins, and it can induce rearrangement of the centrosome and Golgi apparatus relative to the nucleus, indicating a role in microtubule motor-dependent nuclear positioning (Roux et al., 2009). KASH5 is mainly expressed in meiotic cells and can couple to SUN1 and dynein/dynactin-associated complexes, mediating telomere-associated chromosome movement and homologous chromosome pairing (Horn et al., 2013; Morimoto et al., 2012). Taken together, the KASH/Nesprin family should not be viewed as a single class of nuclear-envelope anchoring molecules. Through differential coupling to actin, intermediate filaments and microtubule motor systems, it enables the LINC complex to integrate mechanical inputs from multiple cytoskeletal sources and ultimately convert mechanical signals into intranuclear biological effects, including regulation of nuclear positioning, maintenance of nuclear morphology, chromatin remodelling and control of mechanosensitive gene expression.

### 2.3 Coupling between SUN proteins, the nuclear lamina and chromatin

Whereas KASH/Nesprin proteins select and integrate cytoskeletal mechanical inputs on the cytoplasmic side, SUN proteins constitute the nuclear anchoring and load-bearing end of the LINC complex. The canonical SUN proteins SUN1 and SUN2 localize to the inner nuclear membrane. Their C-terminal SUN domains extend into the perinuclear space and bind KASH domains, whereas their N-terminal domains face the nucleoplasm and can interact with lamin A/C, emerin and other inner nuclear membrane-associated proteins (Crisp et al., 2006; Haque et al., 2006; Haque et al., 2010). This transmembrane topology connects KASH/Nesprin-mediated cytoskeletal input to the nuclear lamina network beneath the inner nuclear membrane, establishing physical continuity between the transnuclear connection interface and intranuclear structures and thereby providing the structural basis for mechanical force transmission from outside the nuclear envelope into the nucleus (Haque et al., 2006; Lombardi et al., 2011; Matsuda and Mofrad, 2022). The nuclear lamina is a filamentous protein network beneath the inner nuclear membrane and is mainly composed of A-type and B-type lamins. Lamin A/C has a key role in maintaining nuclear stiffness, resistance of the nuclear envelope to deformation and the distribution of mechanical stress within the nucleus. Loss of lamin A/C increases nuclear deformability, impairs mechanotransduction and reduces cell survival under mechanical strain, indicating that A-type lamins are important structural components for nuclear mechanical stability and mechanical adaptation (Lammerding et al., 2004). In addition, lamin A expression can increase with tissue or matrix stiffness and participates in matrix stiffness-dependent cell differentiation, suggesting that the nuclear lamina is not a static scaffold but can undergo adaptive regulation in response to the external mechanical environment (Swift et al., 2013). The stability of SUN-lamina connections and the compositional state of the nuclear lamina therefore jointly influence both the efficiency of force transmission into the nucleus and the ability of the nucleus to resist mechanical deformation.

The further effects of mechanical stimulation on chromatin mainly depend on multilayered coupling among the nuclear lamina, inner nuclear membrane proteins and chromatin. The nuclear lamina can spatially associate with lamina-associated domains (LADs) in the genome. LADs are large chromatin domains located near the nuclear periphery and in contact with the nuclear lamina, and are usually associated with more compact chromatin states and relatively low transcriptional activity (Guelen et al., 2008). At the same time, LEM-domain-containing inner nuclear membrane proteins such as emerin, LAP2 and MAN1 can connect with chromatin through barrier-to-autointegration factor (BAF), participating in the positioning and organization of peripheral chromatin (Brachner and Foisner, 2011; Samson et al., 2018). Thus, coupling between the LINC complex and chromatin is more likely to be mediated indirectly through a multilayered interface composed of SUN proteins, the nuclear lamina, LEM-domain proteins and BAF, rather than through universal direct binding between SUN proteins and chromatin. This structural interface provides a basis for transnuclear mechanical stimuli to further influence spatial chromatin organization and transcriptional states, but its dynamic remodelling under force and the transcriptional consequences of that remodelling require further investigation.

### 2.4 Tissue-specific assembly of the LINC complex

The LINC complex is not a single molecular device with fixed composition and connection patterns. Its component combinations and assembly modes vary with cell type, developmental stage and physiological state. Different SUN-domain proteins can pair with specific KASH-domain proteins, thereby forming transnuclear complexes with distinct cytoskeletal connections and nuclear anchoring features (Stroud, 2018). This combinatorial assembly expands the structural diversity of the LINC complex and allows it to adapt to different mechanical tasks, including nuclear positioning, establishment of cell polarity, germ-cell development and chromosome movement. Isoform diversity within the Nesprin family is another important source of LINC structural heterogeneity. SYNE1 and SYNE2 encode not only giant Nesprin-1 and Nesprin-2, but also multiple shorter isoforms with different domain compositions through internal promoters and alternative splicing, with pronounced tissue- and cell-type-specific expression (Ravera et al., 2025). Nesprin-1/2 giant contain both N-terminal actin-binding domains and C-terminal KASH domains, enabling them to connect the actin cytoskeleton to SUN proteins at the outer nuclear membrane. By contrast, some shorter isoforms lack a complete actin-binding domain and may therefore have different cytoskeletal coupling capacities, nuclear envelope localization patterns or regulatory functions (Duong et al., 2014).

Differences in SUN-KASH pairing, Nesprin isoform composition, lamina composition and cytoskeletal adaptor proteins together shape the source, route and strength of mechanical input through the LINC complex. This structural heterogeneity is especially apparent in tissues and cellular states with different mechanical requirements. In skeletal and cardiac muscle, Nesprin-1/2, SUN1/2, lamin A/C and emerin form a stable nucleus-cytoplasm mechanical coupling axis that adapts to cyclic contraction and stretch while maintaining myonuclear positioning and nuclear envelope mechanical stability (De Silva et al., 2023; Zhou et al., 2018). In polarized migrating cells, Nesprin-2G and SUN2 assemble into TAN lines that couple retrograde dorsal actin bundles to the nuclear envelope and drive nuclear movement and centrosome reorientation (Luxton et al., 2010). In meiotic cells, KASH5 connects with SUN1 and the dynein-dynactin complex, transmitting microtubule motor-generated forces to telomere-associated structures and promoting chromosome movement and homologous chromosome pairing (Horn et al., 2013; Morimoto et al., 2012). These examples show that tissue and cellular-state specificity of the LINC complex is not simply a matter of whether a particular component is present or absent. Rather, it is a combinatorial structural adaptation shaped by SUN-KASH pairing, Nesprin isoform expression, cytoskeletal connection mode and the mechanical state of the nuclear lamina. Taken together, this structural heterogeneity provides the structural basis for the first two levels of the proposed continuous framework: distinct cytoskeleton-Nesprin coupling determines the selection of mechanical inputs, whereas SUN-KASH pairing, Nesprin isoform composition and lamina-associated anchoring further influence the routes and distribution of these inputs across the nuclear envelope. The structural organization of the principal cytoskeletal coupling branches and their representative SUN–KASH linkages is summarized in Figure 2.

**FIGURE 2:**
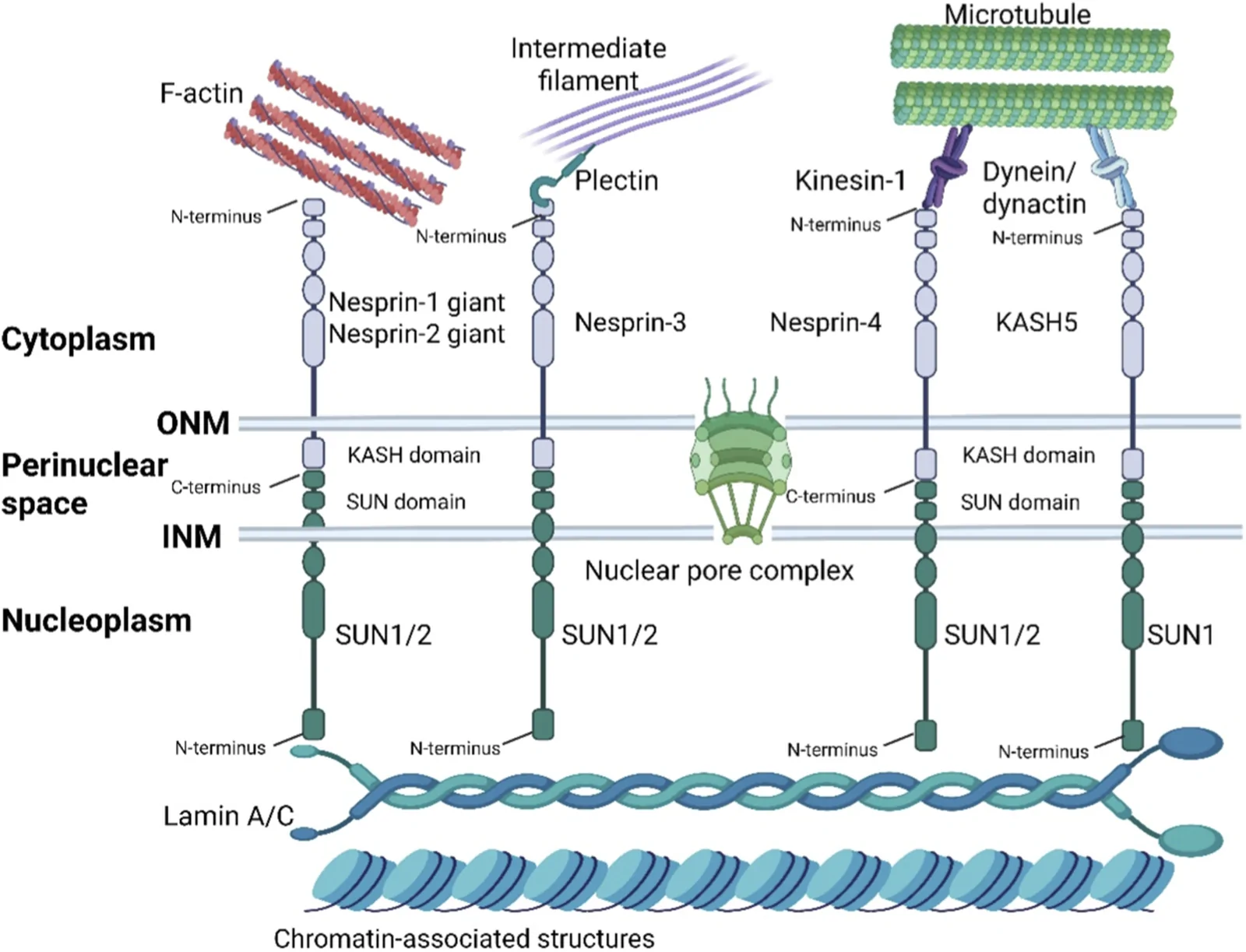
Structural organization and principal cytoskeletal coupling branches of the LINC complex. Nesprin-1 giant and Nesprin-2 giant couple the outer nuclear membrane to F-actin, Nesprin-3 connects it to intermediate filaments through plectin, and Nesprin-4 and KASH5 engage microtubule-associated motor systems through kinesin-1 and dynein/dynactin, respectively. Their C-terminal KASH domains interact with inner nuclear membrane SUN proteins within the perinuclear space, thereby transmitting cytoskeletal forces toward the nuclear lamina and lamina-associated chromatin interfaces. The SUN–KASH pairings shown are representative rather than exclusive. Abbreviations: INM, inner nuclear membrane; ONM, outer nuclear membrane.

## 3 Transnuclear mechanotransduction mediated by the LINC complex

### 3.1 Generation of mechanical input and perinuclear transmission

The mechanical inputs sensed by cells mainly arise from extracellular matrix (ECM) stiffness, spatial confinement, stretch, compression, shear stress and traction forces generated by the cells themselves. ECM elasticity can strongly influence cell behaviour and differentiation, indicating that the ECM is not only a structural scaffold but also an important source of mechanical signals (Saraswathibhatla et al., 2023). These external mechanical states are usually sensed through integrin-mediated cell-ECM adhesions and are converted at focal adhesions into regulated intracellular mechanochemical responses (Kanchanawong and Calderwood, 2023; Young and Reinhart-King, 2023). Focal adhesions are key mechanical conversion nodes between the ECM and the actin cytoskeleton. Talin connects integrins with actin and, upon force-induced stretching, exposes vinculin-binding sites, thereby promoting vinculin recruitment, focal adhesion reinforcement and increased force-transmission efficiency (Wang et al., 2021; Zhao et al., 2022). Meanwhile, the actin-talin-integrin mechanosensitive “molecular clutch” dynamically regulates cellular traction output and downstream signalling strength according to matrix stiffness (Zhao et al., 2022). Within the cell, RhoA-mediated actomyosin contraction further promotes stress-fibre formation and focal adhesion maturation, allowing external mechanical stimuli to be amplified and integrated into tension signals that can propagate along the cytoskeleton (Nishimura et al., 2021). ECM stiffness sensing, integrin adhesion, focal adhesion maturation and actomyosin contraction are therefore not isolated events, but constitute a front-end module for generation and amplification of mechanical input. These tensions are then transmitted toward the perinuclear region through cytoskeletal networks including actin, intermediate filaments and microtubules. Mechanical pulling on integrins can induce cytoskeletal rearrangement, nuclear deformation and changes in intranuclear structure, suggesting the existence of a continuous mechanical coupling pathway between cell-surface adhesion sites and the nucleus (Kalukula et al., 2022; Vivo et al., 2024). Thus, extracellular mechanical signals are sensed, integrated and initially transmitted through the ECM-integrin-focal adhesion-cytoskeleton axis before reaching the perinuclear region, providing the mechanical basis for subsequent LINC-mediated transnuclear force transmission.

### 3.2 LINC-mediated transnuclear force transmission and nuclear mechanical response

The key function of the LINC complex is not simply to fix the nucleus within the cytoplasm, but to maintain effective mechanical coupling between the cytoskeleton and the nucleus. Lombardi et al. disrupted endogenous Nesprin-SUN connections using a dominant-negative KASH construct and found that intracellular force propagation to the nucleus was markedly reduced, accompanied by abnormal organization of perinuclear actin and intermediate filament networks. These findings indicate that an intact LINC complex is required for efficient transmission of cytoskeletal tension to the nucleus (Lombardi et al., 2011). The mechanical load experienced by the nucleus therefore depends not only on the magnitude of external force, but also on the integrity of LINC connections and their coupling with the perinuclear cytoskeleton. LINC-mediated force transmission can directly influence nuclear prestress and patterns of nuclear deformation. In vascular endothelial cells subjected to uniaxial stretch, knockdown of Nesprin-1 altered the initial nuclear shape and increased nuclear strain under stretch (Anno et al., 2012). This result does not mean that Nesprin-1 promotes nuclear deformation. Rather, it suggests that actin continuously applies tension to the nucleus through Nesprin-1, allowing the nucleus to maintain a certain prestressed and geometrically constrained state at rest. When this connection is weakened, the nucleus becomes partially mechanically uncoupled from the actin cytoskeleton, its initial loading state changes, and it shows greater deformation under external stretch (Anno et al., 2012). Thus, the LINC complex is responsible not only for the instantaneous input of mechanical force, but also for setting the basal mechanical state of the nucleus before loading and influencing how external loads are distributed within the nucleus.

The nucleus is also not a completely passive recipient of LINC-transmitted force. Guilluy et al. directly pulled Nesprin-1 on the surface of isolated nuclei using magnetic tweezers and observed that the nucleus rapidly increased its own stiffness under sustained force to resist further deformation. This adaptive nuclear stiffening depended on an intact nuclear lamina and the inner nuclear membrane protein emerin, but not on chromatin or intranuclear actin (Guilluy et al., 2014). These findings indicate that mechanical force applied to the LINC complex can trigger local mechanical adaptation at the nuclear envelope-lamina interface, converting the nucleus from a passive load-bearing object into a response unit capable of adjusting its mechanical properties according to load. This adaptive process helps limit excessive nuclear deformation and modulate stress distribution within the nuclear envelope and lamina. In summary, LINC-mediated transnuclear force transmission is dynamically regulated across different loading states. Constitutive cytoskeletal tension contributes to basal nuclear prestress before external stimulation; acute loading alters nuclear deformation and stress distribution; and sustained or repeated force can induce adaptive stiffening at the nuclear envelope–lamina interface. Thus, the nuclear mechanical state reflects not only the magnitude of the current load but also the pre-existing mechanical state and previous loading history. These changes further influence nuclear envelope tension, the mechanical environment of nuclear pore complexes and the stress state of the lamina–chromatin interface, thereby providing a physical basis for subsequent nucleocytoplasmic transport and chromatin-transcriptional regulation.

### 3.3 Quantitative mechanical parameters of LINC-mediated mechanotransduction

Quantitative measurements indicate that LINC-mediated mechanotransduction spans distinct molecular, nuclear and cellular mechanical scales. At the molecular level, FRET-based tension sensing in living cells estimated that cytoskeleton-generated forces on a mini-Nesprin-2G tension-sensor construct can be as high as approximately 8 pN, corresponding to the upper range of the calibrated sensor and providing a molecular-scale estimate of tension transmitted through a LINC-associated protein (Déjardin et al., 2020). Complementary magnetic-tweezer experiments on isolated nuclei applied approximately 15–35 pN locally to Nesprin-1-associated beads and induced progressive nuclear stiffening, reflected by a maximum reduction of approximately 35% in relative bead displacement after repeated force pulses (Guilluy et al., 2014). These values describe molecular tension or experimentally applied local forces and should not be interpreted as the total force carried by an intact LINC network.

At the whole-nucleus level, microfluidic micropipette aspiration yielded fitted nuclear elastic moduli of 2.7 ± 0.5 kPa in wild-type cells and 1.3 ± 0.3 kPa in lamin A/C-deficient cells (Davidson et al., 2019). Micromanipulation of isolated nuclei further examined nuclear mechanical responses over force ranges of approximately 1–10 nN and deformation length scales of approximately 0.1–10 μm, revealing distinct contributions of chromatin and lamin A/C to nuclear resistance (Stephens et al., 2017). These measurements characterize nuclear material properties rather than the stiffness of the LINC complex itself.

At the cellular and tissue-relevant scale, dynamic tensile loading of mesenchymal stem cells at 3% strain and 1 Hz promoted YAP/TAZ pathway activation, whereas depletion of Nesprin-1 giant markedly attenuated the YAP response. The same study further showed that Nesprin-1 giant depletion reduced nuclear deformation under higher applied tensile strains of 12% and 15%, supporting its role in cytoskeleton-to-nucleus strain transfer (Driscoll et al., 2015). More recently, direct measurements in beating adult cardiomyocytes showed that 11.4% ± 0.4% sarcomere compression at peak systole was accompanied by only a 6.6% ± 0.3% decrease in nuclear length, demonstrating substantial dampening of strain transfer to the nucleus (Amiad Pavlov et al., 2025). Importantly, these studies used different loading geometries, temporal scales and mechanical readouts. A standardized LINC-specific strain-transfer efficiency or prestress value has therefore not yet been established, and molecular tension, applied force, nuclear elastic modulus and cellular strain should not be treated as directly interchangeable quantities. Representative quantitative mechanical parameters across these experimental scales are summarized in Table 1.

**TABLE 1:** Representative quantitative measurements at distinct mechanical scales relevant to LINC-mediated transnuclear mechanotransduction.

Experimental system/loading paradigm	Quantitative parameter	Representative value	LINC-related relevance	Evidence boundary
Living cells; mini-Nesprin-2G FRET tension-sensor construct	Molecular tension on Nesprin	Up to approximately 8 pN	Estimates cytoskeleton-generated tension on a LINC-associated protein	Engineered sensor; 8 pN corresponds to the upper range of the calibrated sensor and does not represent the total force of the native LINC network (Déjardin et al., 2020)
Isolated nuclei; magnetic tweezers applied to Nesprin-1-associated beads	Applied local force and adaptive stiffening	15–35 pN; relative bead displacement decreased by up to approximately 35% after six pulses	Local LINC-directed force induces adaptive nuclear stiffening	Applied force is not equivalent to endogenous total LINC tension; displacement change does not equal a 35% stiffness increase (Guilluy et al., 2014)
Intact cells; microfluidic micropipette aspiration	Fitted nuclear elastic modulus	2.7 ± 0.5 kPa in wild-type cells; 1.3 ± 0.3 kPa in Lmna−/− cells	Demonstrates the contribution of lamin A/C to nuclear load-bearing properties	Nuclear modulus, not LINC stiffness (Davidson et al., 2019)
Isolated nuclei; micromanipulation	Force and deformation assay range	Approximately 1–10 nN; approximately 0.1–10 μm	Defines whole-nucleus mechanical response regimes	Assay/loading range rather than a universal physiological LINC force (Stephens et al., 2017)
Mesenchymal stem cells; cyclic tensile loading	Applied cellular strain	3% strain at 1 Hz for dynamic YAP-response experiments; 12% and 15% applied strain for nuclear-deformation analysis	Nesprin-1 giant-dependent YAP/TAZ activation and nuclear deformation under loading	Applied cellular strain is not equivalent to nuclear strain-transfer efficiency (Driscoll et al., 2015)
Beating adult cardiomyocytes; sarcomere–nucleus strain mapping	Sarcomere-to-nucleus strain coupling	11.4% ± 0.4% sarcomere compression; 6.6% ± 0.3% nuclear shortening	Quantifies physiological dampening of strain transmission to the nucleus	LINC perturbation produced context-dependent changes in coupling; these values do not represent a universal LINC-specific transfer coefficient (Amiad Pavlov et al., 2025)

### 3.4 LINC-mediated nucleocytoplasmic transport and chromatin regulation

Mechanical forces transmitted by the LINC complex to the nuclear envelope-lamina interface can cause nuclear displacement, deformation and changes in nuclear stiffness, and can further influence nucleocytoplasmic transport and chromatin transcriptional states. Current studies support two intranuclear output layers that act on different but interconnected time scales. The first is rapid molecular transport mediated by nuclear deformation and changes in the mechanical state of the nuclear pore complex. The second is remodelling of chromatin conformation and gene expression through the lamina-chromatin connection interface (Alam et al., 2016; Amiad Pavlov et al., 2023; Andreu et al., 2022; Driscoll et al., 2015; Elosegui-Artola et al., 2017; Tajik et al., 2016). At the rapid-response level, LINC-mediated cytoskeleton-nucleus mechanical coupling can influence the nucleocytoplasmic distribution of mechanosensitive transcriptional regulators. Driscoll et al. found that dynamic stretch promoted nuclear entry of YAP in mesenchymal stem cells, whereas knockdown of Nesprin-1 giant reduced transmission of cytoskeletal strain to the nucleus and markedly inhibited stretch-induced YAP nuclear translocation (Driscoll et al., 2015). Elosegui-Artola et al. subsequently revealed a nuclear pore-dependent mechanical regulatory mechanism. When cells on stiff substrates establish stable cytoskeleton-nucleus mechanical connections, cytoskeletal tension flattens the nucleus and pulls on nuclear pore complexes, reducing the mechanical restriction imposed by nuclear pores on molecular transport and thereby increasing the efficiency of YAP nuclear import (Elosegui-Artola et al., 2017). Experiments applying force directly to the nucleus also showed that nuclear loading differentially regulates passive diffusion and receptor-mediated nucleocytoplasmic transport depending on the size of the transported molecule and its affinity for nuclear transport receptors (Andreu et al., 2022). These findings indicate that the LINC complex participates in rapid nuclear translocation of mechanosensitive molecules by transmitting cytoskeletal tension, changing nuclear morphology and altering the mechanical environment of nuclear pores. However, this mechanism does not have the same dependence in all cellular states. In some epithelial cells and epithelial-mesenchymal transition models, Nesprin tension is not required to determine YAP nuclear localization, suggesting that LINC regulation of nucleocytoplasmic transport depends on cell type and mode of mechanical stimulation (Déjardin et al., 2020).

In addition to rapid responses mediated by nuclear pore transport, the LINC complex can transmit mechanical force further to the lamina-chromatin connection interface and thereby influence chromatin conformation directly or indirectly. Tajik et al. applied local force to the cell surface through integrin-bound magnetic beads and observed that mechanical stress could propagate along the actin cytoskeleton, the LINC complex and the lamina-chromatin connection pathway, inducing rapid stretching of specific chromatin regions. The degree of chromatin stretching correlated with transcriptional upregulation of a DHFR reporter gene within that region (Tajik et al., 2016). This study provided direct experimental evidence that force applied at the cell surface can reach chromatin along a continuous structural axis and rapidly influence transcription. However, its conclusions are mainly derived from an engineered chromatin locus and cannot yet be taken to represent a universal response of all endogenous genes. Over longer time scales, the LINC complex also participates in genome-wide transcriptional adaptation induced by matrix mechanics. Alam et al. compared fibroblast transcriptomes under different matrix stiffness conditions and found that disruption of the LINC complex did not simply abolish responses of all stiffness-sensitive genes. Instead, it broadly changed the direction and magnitude of their responses to matrix stiffness, indicating that the LINC complex shapes, rather than solely determines, mechanical-environment-dependent transcriptional programs (Alam et al., 2016). In *Drosophila* larval muscle, loss of LINC complex function also causes chromatin redistribution away from the nuclear periphery, increases binding of transcriptional repressors such as Polycomb and HP1a, and is accompanied by increased repressive histone modifications such as H3K27me3 and H3K9me3 and reduced RNA polymerase II binding (Amiad Pavlov et al., 2023). These results suggest that, in addition to mediating instantaneous force transmission, the LINC complex may maintain spatial contacts between chromatin and the nuclear envelope, limit excessive heterochromatinization and support stable expression of tissue-specific genes.

In summary, LINC-mediated conversion of nuclear responses can be understood as two interconnected output layers. On shorter time scales, cytoskeletal tension transmitted through the LINC complex induces nuclear deformation and changes in nuclear pore transport, thereby regulating nuclear translocation of mechanosensitive molecules such as YAP. On longer time scales, mechanical force can influence spatial chromatin organization, epigenetic state and transcriptional programs through the lamina-chromatin connection interface. It should be emphasized that not all mechanically induced changes in nucleocytoplasmic transport and chromatin can currently be attributed to the LINC complex. Its specific contribution depends on cell type, form of mechanical stimulation, duration of force exposure and the SUN-KASH subtypes involved in assembly. Thus, transnuclear force transmission is not the endpoint of mechanotransduction but is further converted into nuclear responses operating on different time scales: rapid changes in nuclear mechanics and nucleocytoplasmic transport, together with longer-term chromatin remodelling and transcriptional adaptation, constitute the nuclear response conversion level of the proposed framework. These interconnected mechanical, nucleocytoplasmic transport and chromatin responses are illustrated in Figure 3.

**FIGURE 3:**
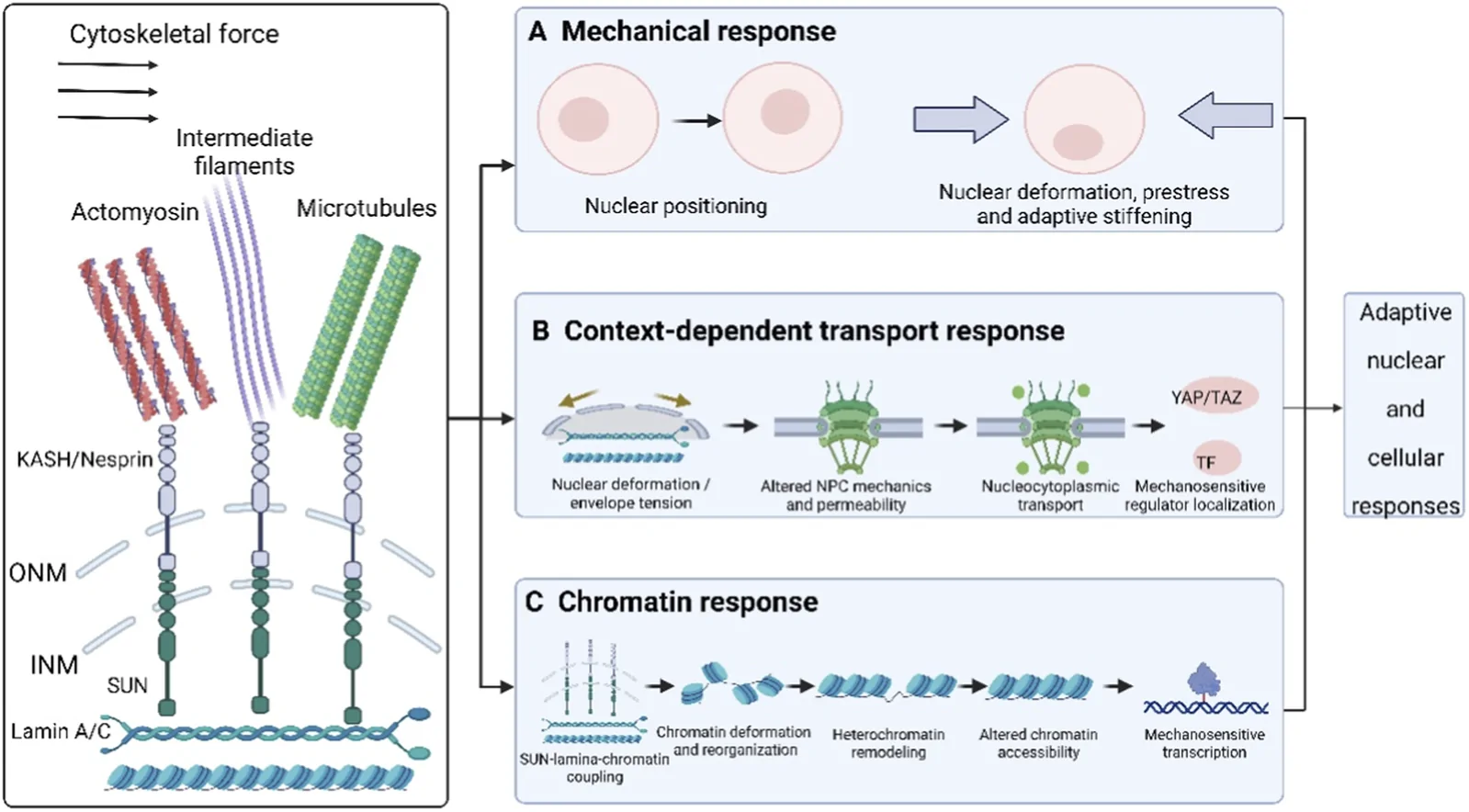
LINC-mediated conversion of cytoskeletal forces into nuclear responses. LINC-mediated force transmission influences three interconnected response routes: **(A)** nuclear positioning, deformation, prestress and adaptive stiffening; **(B)** context-dependent regulation of nuclear pore mechanics, nucleocytoplasmic transport and mechanosensitive regulator localization; and **(C)** chromatin reorganization, altered accessibility and mechanosensitive transcription through the multilayered SUN–lamina–chromatin interface. Together, these routes contribute to adaptive nuclear and cellular responses. Abbreviations: NPC, nuclear pore complex; TF, transcription factor; YAP, Yes-associated protein; TAZ, transcriptional coactivator with PDZ-binding motif.

## 4 Tissue mechanical adaptation mediated by the LINC complex

### 4.1 Nuclear movement, cell migration and morphogenesis during development

During development, the nucleus does not passively remain inside the cell. Instead, it must move and position precisely according to cell-cycle stage, establishment of polarity and direction of migration. Because the nucleus is large and has high mechanical resistance, nuclear movement depends on forces generated by cytoskeletal motors, and these forces must be coupled to the nuclear envelope through the LINC complex (Kirby and Lammerding, 2018; Lee and Burke, 2018; Patel et al., 2026). The core developmental function of the LINC complex is therefore to coordinate cytoskeletal dynamics, relative positioning of the nucleus and centrosome, and whole-cell migration, ensuring that cells reach correct spatial positions and participate in tissue architecture formation (Bone and Starr, 2016; Lima et al., 2024; Luxton et al., 2025). This function is particularly evident in nervous system development. Neuroepithelial progenitors undergo cell-cycle-dependent interkinetic nuclear migration during proliferation, whereas newborn neurons complete radial migration through nucleokinesis. Mouse studies have shown that SUN1 and SUN2 form transnuclear connections with Syne/Nesprin-1/2 and, through Syne-2, interact with dynein/dynactin and kinesin motor systems to maintain coupling between the nucleus and centrosome. Disruption of Sun1/2 or Syne1/2 impairs INM and radial neuronal migration in the cerebral cortex, indicating that the LINC complex is a key effector structure that transmits microtubule motor forces to the nucleus during brain development (Zhang et al., 2009).

Beyond microtubule motor-driven nuclear movement, the LINC complex can regulate nuclear positioning during cell polarization through the actin network. Luxton et al. found that, in polarized migrating fibroblasts, Nesprin-2G and SUN2 form transmembrane actin-associated nuclear lines on the nuclear envelope and move together with retrograde-flowing dorsal actin bundles. Inhibition of Nesprin-2G, SUN2 or actin retrograde flow impairs rearward nuclear movement and centrosome reorientation (Luxton et al., 2010). Although this study used an *in vitro* fibroblast model rather than embryonic tissue, it directly revealed how the LINC complex converts cytoskeletal dynamics into changes in nuclear position and provided important evidence for understanding the mechanical basis of nuclear positioning during development and migration. LINC-dependent nuclear migration also directly participates in formation of layered tissues. During mouse retinal development, Syne-2/Nesprin-2 and SUN1/2 participate in INM of retinal progenitors and photoreceptor nuclear migration, connecting nuclei to dynein/dynactin- and kinesin-associated microtubule motors. Loss of Syne-2 or impaired SUN1/2 function leads to abnormal photoreceptor nuclear positioning, thinning of the outer nuclear layer and reduced retinal electrophysiological function (Yu et al., 2011). These results indicate that LINC-mediated nuclear positioning does not merely affect intracellular organization in individual cells. It can also determine the stratified arrangement of cells within tissues and ultimately influence organ structural and functional maturation.

In summary, by coupling actin and microtubule motors, the LINC complex transmits cytoskeletal dynamics to the nucleus, coordinates nuclear movement, nucleus-centrosome positioning and cell polarity, and thereby supports ordered layering and morphogenesis in tissues such as the cerebral cortex and retina. The LINC complex therefore not only maintains the spatial position of the nucleus, but also serves as an important mechanical node that converts subcellular nuclear dynamics into tissue-scale developmental outcomes.

### 4.2 Nuclear mechanical protection and functional adaptation in skeletal and cardiac muscle

Skeletal and cardiac muscle are highly mechanosensitive tissues that are chronically exposed to cyclic contraction, stretch and compression. Unlike most mononuclear cells, skeletal muscle fibres contain many myonuclei distributed at the cell periphery, whereas cardiomyocyte nuclei are continuously subjected to forces generated by sarcomere contraction and cell deformation (Castellano et al., 2025; Murphy and Li, 2026; Roman et al., 2021) These tissues must therefore maintain stable connections between the nuclear envelope and cytoskeleton, while also coordinating myonuclear position, nuclear morphology and nuclear responses induced by mechanical load. Through connections among actin, microtubules, sarcomere-associated structures and the nuclear envelope-lamina interface, the LINC complex establishes an important physical link between myonuclear positioning and mechanical load responses (De Silva, Fan, Kang, Shanahan and Zhang, 2023; Heffler et al., 2020; Wang et al., 2018). In skeletal muscle, this transmembrane force-transmission structure is a core molecular basis for ordered spatial arrangement of myonuclei. Myonuclei are not randomly distributed. They must maintain relatively uniform internuclear spacing and form specific synaptic nuclear clusters beneath neuromuscular junctions (Bagley et al., 2023; Roman and Gomes, 2018; Stroud et al., 2017). Mouse genetic studies show that deletion of the KASH domain of Syne-1 disrupts anchoring of synaptic myonuclei and disturbs the arrangement of non-synaptic myonuclei. Concurrent loss of Syne-1 and Syne-2 causes more severe myonuclear positioning abnormalities and is accompanied by abnormal diaphragmatic innervation and respiratory dysfunction in newborn mice (Zhang X. et al., 2007). SUN1 and SUN2 have partially overlapping roles in this process. As the number of functional Sun1/2 alleles decreases, defects in synaptic myonuclear anchoring progressively worsen, and double loss of Sun1/2 disrupts both Nesprin-1 localization at the myonuclear membrane and the ordered distribution of non-synaptic myonuclei (Lei et al., 2009). These findings indicate that SUN-KASH connections not only anchor myonuclei within muscle fibres, but also maintain the spatial organization of synaptic and non-synaptic nuclei, providing a structural basis for neuromuscular junction formation, motor innervation and muscle-fibre function.

In addition to maintaining myonuclear position, the LINC complex participates in converting mechanical inputs generated by muscle-fibre contraction into adaptive nuclear responses. In *Drosophila* larval striated muscle, loss of the LINC components Klar or Klaroid causes DNA replication among different myonuclei in the same muscle fibre to become desynchronized, leading to abnormal and persistent endoreplication and altered chromatin-binding profiles of RNA polymerase II (Wang et al., 2018). Weakening integrin connections between muscle fibres and tendons produces similar abnormalities in myonuclear DNA content, indicating a functional link between mechanical input experienced by muscle fibres and myonuclear growth and genome regulation (Wang et al., 2018). Although this evidence comes from a *Drosophila* model, it suggests that in multinucleated muscle fibres the LINC complex may help different myonuclei synchronously sense a shared cytoplasmic mechanical environment, thereby coordinating muscle-fibre growth and functional adaptation.

The mechanical connection formed jointly by the LINC complex and nuclear lamina can also limit excessive effects of mechanical load on myonuclear chromatin state. In a human skeletal myotube model, acute mechanical stretch changes chromatin accessibility and gene expression. When lamin A/C is absent, stretch-induced chromatin opening is markedly enhanced and stress- and DNA damage-related gene pathways are more strongly activated (Jabre et al., 2025). This result shows that the nuclear lamina not only determines nuclear stiffness, but can also buffer the effects of mechanical stress on chromatin, helping muscle cells maintain transcriptional programs and genome state while receiving mechanical stimulation. It should be noted that this study directly examined lamin A/C rather than isolated interference with core LINC components. It is therefore more appropriately used to illustrate the intranuclear protective role downstream of the continuous LINC-lamina mechanical axis. In myocardium, the LINC complex maintains cardiomyocyte nuclear position and morphology and also participates in mechanical load-induced gene-expression responses. Cardiomyocyte-specific double knockout of Nesprin-1 and Nesprin-2 causes abnormal nuclear position, shape and chromatin distribution. Under mechanical stretch, mutant cardiomyocytes fail to upregulate mechanosensitive genes such as Egr1, Iex1, c-Fos, c-Jun and c-Myc to the same extent as control cells (Banerjee et al., 2014). Single knockout of Nesprin-1 or Nesprin-2 causes only partial defects in nuclear morphology and gene response, whereas double knockout produces more pronounced structural and functional cardiac abnormalities, indicating a degree of functional redundancy in myocardium (Banerjee et al., 2014). Thus, Nesprin-1/2-mediated nuclear-sarcomeric mechanical coupling not only helps maintain spatial stability of cardiomyocyte nuclei, but also provides an important condition for cardiomyocytes to integrate mechanical load and initiate adaptive transcriptional responses.

In summary, the physiological functions of the LINC complex in skeletal and cardiac muscle mainly include maintenance of myonuclear anchoring, spacing and tissue-specific distribution; transmission of mechanical load generated by muscle contraction and tissue stretch to the nuclear envelope-lamina interface; and coordination of chromatin, DNA replication and gene-expression responses related to mechanical load. In this way, the LINC complex allows myonuclei to stably withstand continuous mechanical action while adjusting nuclear programs in response to changing tissue load. When this mechanical connection is damaged, normal adaptive mechanisms may be converted into myonuclear mispositioning, nuclear structural injury and transcriptional imbalance, ultimately producing selective vulnerability of skeletal and cardiac muscle. Thus, the tissue-level outcome of LINC-mediated mechanical information depends on whether the preceding levels remain coordinately matched: when mechanical input, transmembrane force distribution and nuclear responses are appropriately matched, they support normal tissue development and mechanical adaptation; when this matching is disrupted, it may create tissue-specific vulnerability and ultimately lead to disease phenotypes.

## 5 LINC complex abnormalities and mechanotransduction-related disease

### 5.1 Mechanical coupling imbalance and abnormal nuclear responses

The common pathological basis of LINC complex-related diseases should not be understood simply as structural fragility caused by loss of a particular nuclear envelope protein. It is more accurately described as distortion of information transmission along the continuous cytoskeleton-nuclear envelope-lamina mechanical axis. Under normal conditions, the LINC complex not only transmits cytoskeleton-generated force to the nucleus, but also cooperates with the nuclear lamina to mediate, or maintain, nuclear prestress, restrict abnormal deformation and convert mechanical input into adaptive changes in nuclear pore transport, chromatin conformation and transcriptional programs (Elosegui-Artola et al., 2017; Kirby and Lammerding, 2018; Nava et al., 2020; Tajik et al., 2016). Therefore, when SUN-KASH connections, Nesprin-mediated cytoskeletal coupling, lamin A/C, emerin or other lamina-associated components are impaired, pathology does not simply manifest as a failure of force to enter the nucleus. Instead, the magnitude, direction and duration of mechanical input, as well as the form of nuclear response, shift systematically. This shift constitutes the first shared mechanism through which LINC complex abnormalities progress from molecular defects to tissue disease.

Altered mechanical coupling is the starting point of this pathological cascade, but it is not equivalent to a complete interruption of mechanical signalling. Disruption of SUN-KASH interaction markedly weakens the transmission of cytoskeletal deformation to the nucleus and disturbs the ordered organization of perinuclear actin and intermediate filament networks (Lombardi et al., 2011). This finding suggests that LINC complex defects first alter the mechanical boundary conditions surrounding the nucleus. The nucleus may lose stable prestress constraints or may experience abnormally distributed local stress under external stretch, shear or contractile load. In other words, an impaired LINC axis does not merely reduce incoming force. It reshapes how force reaches the nucleus, where it acts and how long it persists. The same category of molecular defect may therefore produce different readouts in different cells, including disordered nuclear positioning, abnormal nuclear morphology, concentrated nuclear envelope stress or sluggish mechanical responses. This helps explain why LINC-related phenotypes are highly dependent on cytoskeletal architecture and the tissue mechanical environment.

After mechanical input is mis-transmitted, the buffering and adaptive capacity of the nuclear envelope-lamina interface becomes a second node that determines whether injury is amplified. Loss of lamin A/C reduces nuclear stiffness, makes nuclei more susceptible to excessive deformation under stretch and weakens mechanosensitive transcriptional responses such as NF-kappaB activation (Lammerding et al., 2004). Emerin participates in adaptive nuclear stiffening after force exposure, and its loss can disrupt mechanically induced gene expression and increase apoptosis under sustained stretch even when global nuclear mechanical properties are not necessarily markedly changed (Guilluy et al., 2014; Lammerding et al., 2005). These data indicate that LINC-related diseases cannot be explained by a single concept of increased nuclear envelope fragility. More precisely, pathology includes two interrelated but non-identical processes: reduced ability of nuclear structures to bear mechanical load, and impaired ability of the nucleus to convert mechanical stimulation into adaptive biological responses. The former makes the nucleus more vulnerable to injury, whereas the latter prevents cells from initiating appropriate protective and remodelling programs even when they sense mechanical change. This impairment of nuclear response further manifests as imbalance in spatial chromatin organization and transcriptional regulation. Importantly, LINC complex abnormalities do not uniformly suppress all mechanosensitive genes. They alter the direction, magnitude and selectivity of mechanical responses. Disruption of the LINC complex broadly reshapes the expression patterns of matrix-stiffness-sensitive genes (Alam et al., 2016). In skeletal muscle models, loss of LINC function is associated with redistribution of perinuclear chromatin, increased repressive histone modifications and reduced RNA polymerase II binding (Amiad Pavlov et al., 2023). In human myotubes lacking lamin A/C, mechanical stretch leads to excessive chromatin opening and abnormal activation of stress- and DNA damage-related pathways (Jabre et al., 2025). In cardiomyocytes, loss of Nesprin-1/2 weakens mechanical load-induced immediate early gene expression (Banerjee et al., 2014). These apparently different results point to a higher-level pathological concept: when the LINC-lamina axis is impaired, intranuclear mechanical responses do not simply disappear. Instead, response patterns become distorted, manifesting as reduced responses, excessive chromatin opening, ectopic activation, delayed responses or changes in direction.

The shared pathological cascade in LINC complex-related disease can therefore be summarized as distortion of cytoskeleton-nucleus mechanical coupling, followed by failure of nuclear envelope-lamina load distribution and adaptive buffering, and then abnormal remodelling of chromatin organization, nucleocytoplasmic transport and mechanosensitive transcriptional programs. This framework also provides a basis for explaining tissue selectivity. Skeletal and cardiac muscle are chronically exposed to cyclic contraction and stretch and therefore rely heavily on the integrity of this continuous mechanical axis. By contrast, non-muscle phenotypes such as neurological disease, premature ageing and tumour progression may more often reflect disruption of specific LINC functions in nuclear positioning, chromatin homeostasis, nuclear pore transport or cell migration.

### 5.2 Tissue-selective vulnerability in EDMD and cardiomyopathy

The selective vulnerability of skeletal and cardiac muscle in LINC complex-related diseases is more accurately understood as the combined result of cyclic mechanical loading and tissue-specific requirements for nucleus-cytoskeleton connections, rather than simply as a consequence of greater mechanical force. Skeletal muscle must maintain uniform distribution of myonuclei within multinucleated fibres and anchoring of synaptic nuclei beneath neuromuscular junctions. The heart must maintain nuclear morphology, perinuclear cytoskeletal organization and load-induced gene responses during continuous contraction-relaxation cycles. Tissue-specific expression of Nesprin isoforms and the important role of Nesprin-1α2 in striated muscle further indicate that different tissues do not depend on LINC components in the same way (Duong et al., 2014; Storey and Fuller, 2022; Wang et al., 2018). Thus, persistent load amplification is a useful framework for explaining tissue selectivity in EDMD and cardiomyopathy, but it is not sufficient as the only mechanism. Component expression, isoform composition and the mechanical tasks of different tissues also jointly determine disease phenotypes.

Human genetic evidence supports associations between LINC-related variants and EDMD or EDMD-like myopathies, but the terminology should distinguish confirmed pathogenic mutations, candidate variants and potential disease modifiers. In patients with EDMD or EDMD-like disease, C-terminal variants in SYNE1 and SYNE2 are associated with abnormal nuclear morphology, altered nuclear envelope localization of emerin or SUN2, and impaired nucleus-cytoskeleton connections (Zhang Q. et al., 2007). Rare SUN1/SUN2 variants have also been identified in patients with EDMD or related myopathies. Functional experiments show that some variants can disrupt nucleus-cytoskeleton connections and myonuclear organization and may influence disease severity in the background of other nuclear envelope protein variants (Meinke et al., 2014; Storey and Fuller, 2022). These findings indicate that EDMD is not a disease caused by a single nuclear envelope protein defect. Similar muscle-cardiac phenotypes can arise from damage to different nodes within the LINC complex and its lamina-associated connection network. At the same time, because some studies include small numbers of cases and variant evidence varies in strength, SYNE1/2 or SUN1/2 variants should be described as associated or possibly pathogenic when appropriate, avoiding direct equivalence between all rare variants and confirmed pathogenic mutations (Meinke et al., 2014; Storey and Fuller, 2022; Zhang Q. et al., 2007).

In skeletal muscle, the most consistent experimental readout is abnormal myonuclear anchoring and spatial organization. Impaired Syne1/Syne2 or SUN1/SUN2 function causes defects in synaptic myonuclear anchoring and disordered non-synaptic myonuclear arrangement, and in severe models is accompanied by diaphragmatic innervation and respiratory abnormalities (Lei et al., 2009; Zhang X. et al., 2007). Loss of Nesprin-1α2 also reduces postnatal viability and causes abnormal myonuclear positioning (Stroud et al., 2017). More directly, mice lacking the Nesprin-1 KASH domain develop progressive muscle weakness, abnormal gait and kyphosis, resembling EDMD (Puckelwartz et al., 2009). Together, these models support a pathological chain of myonuclear mispositioning, load-bearing imbalance and reduced muscle-fibre function. However, the severity of myopathy differs among Syne1 models, suggesting that myonuclear mispositioning may be an important early pathological intermediate, but may not by itself explain the full muscular dystrophy phenotype. The effects of mutations on different Nesprin isoforms, LINC assembly modes and cytoskeletal connections are also critical (Puckelwartz et al., 2009; Stroud et al., 2017; Zhang X. et al., 2007).

In myocardium, LINC defects affect nuclear structure, cardiac conduction and mechanically adaptive transcription. Cardiomyocyte-specific double knockout of Nesprin-1 and Nesprin-2 causes cardiomyopathy, abnormal nuclear position and morphology, and impaired immediate early gene expression induced by mechanical stretch (Banerjee et al., 2014). Rare C-terminal variants in Nesprin-1 encoded by SYNE1 have been identified in patients with dilated cardiomyopathy, and functional experiments show that these variants can cause abnormal nuclear morphology and reduce nuclear envelope localization of lamin A/C and SUN2 (Zhou et al., 2017). Another study reported that a patient carrying the Nesprin-1alpha R374H variant had severe dilated cardiomyopathy, patient-derived cells showed abnormalities in LINC components, and corresponding KASH-domain-deficient mice developed progressive cardiomyopathy and conduction defects (Puckelwartz et al., 2010). These findings indicate that the LINC complex participates both in distributing perinuclear stress generated by cardiac contraction and in converting mechanical load into adaptive gene programs.

Studies of LMNA-related cardiomyopathy reveal a profound paradox in mechanical transmission: the pathological mechanism is not simply insufficient mechanical input into the nucleus. Under physiological conditions, LINC-mediated nucleus-cytoskeleton coupling is important for maintaining nuclear spatial positioning and responses to mechanical load (Almonacid et al., 2019; Lee and Burke, 2018). However, when lamin A/C defects substantially reduce nuclear load-bearing capacity, microtubule forces transmitted through the same pathway can concentrate stress locally at the nuclear envelope and drive pathological nuclear structural injury. In this setting, blocking microtubule forces or weakening this mechanical coupling can reduce nuclear damage and improve cardiac function (Amiad Pavlov et al., 2025). In addition, in LMNA-deficient cardiomyocytes, mechanical stress input through the LINC complex can induce abnormal genome-lamina interactions and three-dimensional chromatin rearrangement (Shen et al., 2026). Thus, in tissues exposed to high mechanical load, pathological phenotypes reflect the coexistence of insufficient physiological mechanotransduction and pathological local stress overload. The former causes failure to initiate adaptive transcriptional programs, whereas the latter accelerates physical damage to the nuclear envelope and chromatin instability.

Although EDMD and cardiomyopathy exemplify the vulnerability of mechanically loaded striated muscle to LINC dysfunction, LINC-related pathology extends beyond muscle and produces distinct disease phenotypes in other cellular and mechanical contexts.

### 5.3 LINC dysfunction in neurological disease, premature ageing and tumour progression

In neurological disease, mutations in the LINC component SYNE1 were initially identified as a cause of autosomal-recessive cerebellar ataxia (Gros-Louis et al., 2007). Subsequent multicentre analyses showed that SYNE1-associated ataxia may also involve non-cerebellar neurological manifestations, indicating substantial phenotypic heterogeneity (Synofzik et al., 2016). Consistent with these genetic findings, Sun1-deficient mice exhibit reduced cerebellar volume, abnormal positioning and maturation of Purkinje cells, and impaired dendritic and synaptic organization (Wang et al., 2015). These observations suggest that defective LINC-dependent nuclear positioning and cytoskeletal coupling may interfere with neuronal morphogenesis and circuit function. Nevertheless, direct measurements of force transmission through neuronal LINC complexes remain limited, and the contribution of altered mechanical coupling to human neurological disease requires further investigation.

In progeroid syndromes, the available evidence supports a mechanistic link between abnormal nuclear lamina composition and LINC dysfunction. Abnormal accumulation of SUN1 contributes to disease phenotypes in cellular and mouse models of progeroid laminopathy, whereas SUN1 reduction can alleviate nuclear abnormalities, cellular senescence and disease-associated phenotypes (Chen et al., 2012; Puente et al., 2011). More recent work showed that farnesylated prelamin A or progerin interferes with nesprin-2–SUN2 LINC-complex function, reduces the mobility of these proteins and impairs actin-dependent cell polarization (Lio et al., 2026). These findings indicate that premature ageing may result not only from altered nuclear-lamina integrity but also from disruption of LINC-mediated cytoskeleton–nucleus coupling and force transmission.

LINC dysfunction also contributes to tumour-cell migration and invasion in a context-dependent manner. During confined migration through three-dimensional matrices, nesprin-2 and Lis1 help couple the nucleus to the centrosome and coordinate the positioning of MT1-MMP-containing compartments, thereby supporting local matrix degradation and tumour-cell invasion (Infante et al., 2018). By contrast, depletion of SUN1 and SUN2 in breast carcinoma cells migrating along aligned fibrillar extracellular matrix increased nuclear deformation and cell motility through enhanced F-actin formation and MKL1-dependent transcriptional responses (Sharma et al., 2021). In melanoma cells, RAC1-dependent alterations of the LINC complex and nuclear architecture have also been linked to increased invasiveness (Colón-Bolea et al., 2020). These apparently divergent effects indicate that LINC disruption does not uniformly suppress or promote tumour invasion. Instead, its outcome likely depends on the cytoskeletal branch involved, the degree of matrix confinement, nuclear mechanical properties and the mechanical demands of the tumour microenvironment.

Collectively, these examples broaden the disease spectrum of LINC dysfunction beyond striated muscle. They also support the view that LINC-associated disease is not simply caused by a general loss of complex activity. Rather, tissue-specific phenotypes may arise from an imbalance in particular SUN-KASH connections, altered coupling to distinct cytoskeletal systems and context-dependent conversion of mechanical inputs into nuclear and transcriptional responses.

In summary, EDMD and related cardiomyopathies are typical tissue-specific readouts of imbalance in the LINC-lamina mechanical axis in striated muscle. Skeletal muscle pathology more prominently involves myonuclear anchoring, internuclear spacing and neuromuscular-junction-associated nuclear organization, whereas cardiac pathology more prominently involves perinuclear load distribution, conduction-system involvement and impaired mechanically induced transcription. Both share a basic pathological framework of tissue-specific assembly defects, amplification by cyclic load, and imbalance of nuclear structure and nuclear responses. At the same time, studies of neurological disease, premature ageing and cancer show that the consequences of LINC complex abnormalities are not limited to high-load tissues, but depend on the specific requirements of different cells for nuclear positioning, nuclear envelope-chromatin homeostasis and nuclear deformability. The major LINC-mediated force-transmission modules, nuclear outputs and disease-relevant consequences are summarized in Table 2.

**TABLE 2:** Key LINC-mediated mechanotransduction modules and their physiological and disease-related consequences.

Mechanotransduction module	Main molecular components	Mechanical role	Physiological output	Disease-related consequence
SUN-KASH core linkage	SUN1/2; KASH/Nesprin proteins	Forms a force-bearing bridge across the nuclear envelope and maintains cytoskeleton–nucleus mechanical continuity	Nuclear positioning, nuclear shape maintenance and basal nuclear prestress	Mechanical uncoupling and reduced force transmission to the nucleus (Gurusaran and Davies, 2021; Jahed et al., 2021; Lombardi et al., 2011; McGillivary et al., 2023; Sosa et al., 2012)
Actin–Nesprin branch	Nesprin-1/2 giant; actin filaments; TAN-line-associated SUN2/Nesprin-2G	Couples actomyosin tension and retrograde actin flow to the nuclear envelope	Nuclear movement, cell polarity, centrosome reorientation and mechanosensitive YAP transport	Impaired nuclear positioning, altered nuclear deformation and abnormal mechanosensitive transcription (Anno et al., 2012; Driscoll et al., 2015; Elosegui-Artola et al., 2017; Luxton et al., 2010)
Intermediate filament branch	Nesprin-3; plectin; keratin or other intermediate filaments	Links the outer nuclear membrane to intermediate filament networks and organizes perinuclear cytoskeletal architecture	Nuclear mechanical support and flow-induced cellular polarization	Perinuclear cytoskeletal disorganization and altered nuclear–cytoskeletal integrity (Morgan et al., 2011; Wilhelmsen et al., 2005)
Microtubule-associated branch	Nesprin-4; KASH5; dynein/dynactin; kinesin-associated systems	Couples microtubule motor forces to nuclear or chromosome movement	Nuclear positioning, retinal and neuronal development, meiotic chromosome motion	Nuclear positioning defects; pathological microtubule-driven nuclear damage when nuclear load-bearing capacity is reduced (Amiad Pavlov et al., 2025; Horn et al., 2013; Morimoto et al., 2012; Roux et al., 2009; Yu et al., 2011; Zhang et al., 2009)
Nuclear lamina–chromatin interface	SUN proteins; lamin A/C; emerin; LEM-domain proteins; BAF; chromatin	Receives LINC-transmitted forces and converts them into nuclear mechanical and chromatin responses	Adaptive nuclear stiffening, chromatin organization and transcriptional regulation	Nuclear fragility, distorted chromatin responses, progeroid phenotypes and laminopathy-related dysfunction (Brachner and Foisner, 2011; Crisp et al., 2006; Guelen et al., 2008; Guilluy et al., 2014; Haque et al., 2006; Haque et al., 2010; Jabre et al., 2025; Lammerding et al., 2005; Lammerding et al., 2004; Nava et al., 2020; Samson et al., 2018; Swift et al., 2013)
Striated muscle LINC axis	Nesprin-1/2; Nesprin-1α2; SUN1/2; lamin A/C; emerin	Maintains myonuclear anchoring, perinuclear load distribution and mechanically induced gene responses	Skeletal muscle nuclear spacing, neuromuscular-junction-associated nuclear organization and cardiomyocyte adaptation	EDMD-like myopathy, dilated cardiomyopathy, conduction abnormalities and impaired adaptive transcription (Banerjee et al., 2014; Lei et al., 2009; Meinke et al., 2014; Puckelwartz et al., 2009; Puckelwartz et al., 2010; Stroud et al., 2017; Zhang Q. et al., 2007; Zhang X. et al., 2007; Zhou et al., 2017)
Context-dependent disease output	LINC complex and associated nuclear envelope–lamina network	Balances mechanical coupling, local load distribution and nuclear response conversion	Tissue-specific mechanical adaptation	Mechanical uncoupling, local stress overload, neurological disease, premature ageing and tumour invasion (Amiad Pavlov et al., 2025; Chen et al., 2012; Colón-Bolea et al., 2020; Dupré et al., 2007; Puente et al., 2011; Storey and Fuller, 2022)

Abbreviations: BAF, barrier-to-autointegration factor; EDMD, Emery–Dreifuss muscular dystrophy; LEM, LAP2–emerin–MAN1; TAN, lines, transmembrane actin-associated nuclear lines.

## 6 Discussion and perspectives

Current research has expanded our understanding of the LINC complex from a static nucleus-cytoskeleton connector to a key dynamic platform that regulates transnuclear mechanical information transfer. Different SUN-KASH combinations couple to actin, intermediate filament or microtubule-related systems, giving the LINC complex the ability to select and integrate different mechanical inputs. The assembly state of the complex and its spatial organization at the nuclear envelope further influence the efficiency of transnuclear force transmission and local load distribution (Lombardi et al., 2011; McGillivary et al., 2023). Mechanical force transmitted through the LINC complex not only regulates nuclear positioning, morphology and prestress, but also affects nuclear pore transport, chromatin conformation and mechanosensitive transcriptional programs through the nuclear envelope-lamina interface (Elosegui-Artola et al., 2017; Guilluy et al., 2014; Tajik et al., 2016). These findings support a continuous framework composed of mechanical input selection, transmembrane force distribution, nuclear response conversion, and tissue adaptation or disease output. Within this framework, the LINC complex becomes a central hub connecting subcellular structural mechanics with tissue functional regulation.

This framework builds on established research on LINC-mediated force transmission and nuclear mechanosensing. Experimental work by Lombardi et al. showed that disruption of SUN-KASH/LINC coupling impairs force transmission from the cytoskeleton to the nucleus (Lombardi et al., 2011), whereas Kirby and Lammerding further emphasized that the nucleus itself can function as a mechanosensitive compartment, in which the nuclear envelope, lamina and chromatin participate in the conversion of mechanical signals into intranuclear structural and functional responses (Kirby and Lammerding, 2018). Building on these foundations, the present review further integrates these established concepts into a continuous cross-scale sequence linking mechanical input selection and transmembrane force distribution to nuclear response conversion and, ultimately, tissue adaptation or disease outcome. Such integration helps explain different forms of force-transmission imbalance in LINC-related diseases. Impaired SUN-KASH connections can reduce the overall coupling efficiency between the cytoskeleton and the nucleus, making mechanical stimuli difficult to convert accurately into adaptive nuclear responses (Lombardi et al., 2011). At the same time, when some LINC connection branches remain intact but nuclear load-bearing capacity is reduced, forces generated by perinuclear microtubules may be transmitted through the LINC axis and concentrated at local regions of the nuclear envelope, producing pathological stress overload and accelerating nuclear structural injury (Amiad Pavlov et al., 2025). Accordingly, LINC dysfunction may manifest as either global coupling insufficiency or local stress overload, depending on the affected cytoskeleton–LINC branch and the load-bearing capacity of the nucleus. Disease phenotypes therefore depend on whether the LINC complex can dynamically match the sources of mechanical input, transmembrane transmission routes and nuclear load-bearing capacity, rather than simply on whether mechanical force is increased or decreased in absolute terms.

Tissue selectivity can be further understood as a specific match between tissue mechanical tasks and LINC assembly features. Skeletal and cardiac muscle are exposed to chronic cyclic mechanical load and depend, respectively, on ordered myonuclear distribution, neuromuscular-junction-associated nuclear anchoring, perinuclear structural stability and mechanically induced transcriptional responses. They therefore have high dependence on LINC-axis integrity (Banerjee et al., 2014; Lei et al., 2009; Zhang X. et al., 2007). At the same time, Nesprin isoform composition, functional redundancy among SUN proteins, dominant cytoskeletal connection branches and nuclear geometry jointly determine the compensatory capacity and pathogenic thresholds of different cells under LINC defects. Together, the interaction among tissue mechanical tasks, LINC assembly features and nuclear load-bearing capacity may help explain both selective vulnerability in EDMD and cardiomyopathy and differences in tissue preference, disease severity and clinical phenotype among LINC-related variants (Meinke et al., 2014; Zhang Q. et al., 2007).

Existing evidence spans isolated nuclei, living cells, animal models and patient variant studies, and supports key roles for the LINC complex in mechanical input integration, transnuclear transmission and regulation of nuclear responses. This mechanistic interpretation is supported by complementary evidence from different experimental levels. Isolated-nucleus and cell-based studies primarily define direct mechanical and cellular mechanisms, animal models further test their *in vivo* tissue-level consequences, whereas patient-associated and human genetic studies mainly establish relevance to human disease phenotypes. However, different studies use substantially different types, intensities and durations of mechanical stimulation, as well as different evaluation metrics, which limits direct comparison across models. The next stage of research should standardize mechanical stimulation parameters in tissue-specific models and simultaneously track mechanical input, LINC force-transmission state, nuclear responses and disease phenotypes, so that causal order and temporal relationships among these steps can be clarified. Representative experimental paradigms, causal perturbations, principal readouts and interpretive boundaries are summarized in Table 3.

**TABLE 3:** Representative experimental evidence linking LINC-mediated mechanical input to nuclear mechanical and transcriptional responses.

Experimental system and mechanical paradigm	LINC-related perturbation or structural requirement	Primary readout	Mechanistic inference	Evidence boundary
Living-cell local force-propagation assays	Dominant-negative KASH disruption of Nesprin–SUN binding	Nuclear strain; perinuclear cytoskeletal organization	An intact SUN-KASH linkage is required for efficient cytoskeleton-to-nucleus force transmission	Acute cell-based assays do not capture long-term tissue adaptation (Lombardi et al., 2011)
Vascular endothelial cells under uniaxial stretch	Nesprin-1 knockdown	Initial nuclear shape and stretch-induced nuclear strain	Nesprin-1 contributes to basal nuclear prestress and constrains deformation under load	Cell-type- and loading-mode-specific (Anno et al., 2012)
Isolated nuclei; Nesprin-1 magnetic-tweezer pulling	Dependence on lamin A/C and emerin	Force-induced nuclear stiffening	LINC-applied force can trigger adaptive stiffening at the nuclear envelope–lamina interface	Isolated nuclei exclude cytoplasmic feedback and tissue context (Guilluy et al., 2014)
Mesenchymal stem cells under dynamic stretch	Nesprin-1 giant knockdown	Strain transfer to the nucleus; YAP nuclear translocation	LINC-mediated nuclear deformation supports rapid mechanosensitive nuclear import	Focused on YAP and one cell model (Driscoll et al., 2015)
Stiffness-controlled cells or direct nuclear loading	Intact cytoskeleton–nucleus coupling; altered nuclear shape/NPC mechanics	Passive and receptor-mediated transport; YAP import	Nuclear force and deformation regulate transport through mechanically sensitive nuclear pores	The contribution of individual SUN-KASH pairs was not fully resolved (Andreu et al., 2022; Elosegui-Artola et al., 2017)
Integrin-bound magnetic-bead force	Interrogation of the actin–LINC–lamina–chromatin axis	Chromatin stretching; DHFR reporter transcription	Mechanical force can propagate from adhesion sites to chromatin and rapidly modulate transcription	Evidence is based largely on an engineered reporter locus (Tajik et al., 2016)
Fibroblasts on stiffness-controlled matrices; *Drosophila* muscle	LINC disruption or loss of Klar/Klaroid	Transcriptome; peripheral chromatin; repressive histone marks; RNA polymerase II	LINC integrity shapes stiffness-dependent transcription and chromatin organization	Cross-model heterogeneity limits direct comparison (Alam et al., 2016; Amiad Pavlov et al., 2023)
Epithelial and epithelial–mesenchymal transition models	Nesprin tension perturbation	YAP nuclear localization	LINC dependence of YAP localization is not universal	Negative/context-dependent evidence; response varies with cell state and stimulus (Déjardin et al., 2020)

This table summarizes representative experimental studies that collectively illustrate complementary levels of evidence for LINC-mediated mechanotransduction. The “Evidence boundary” column indicates the principal constraint affecting causal inference or generalizability of each study. Abbreviations: DHFR, dihydrofolate reductase; LINC, linker of nucleoskeleton and cytoskeleton; NPC, nuclear pore complex; YAP, Yes-associated protein.

This research path can support a series of testable hypotheses on mechanisms of tissue vulnerability, molecular disease stratification and evaluation of intervention effects. First, the phenotype of the same LINC component variant is expected to be influenced by the dominant cytoskeletal connection branch in different tissues, so functional assessment of patient variants should preferentially be performed in the relevant tissue context and under physiological mechanical load. Second, disease states dominated by mechanical uncoupling and those dominated by local stress overload may require interventions in different directions, and simple global restoration or weakening of nucleus-cytoskeleton connections is unlikely to cover all pathological types. Third, abnormalities in mechanosensitive nucleocytoplasmic transport, chromatin accessibility and immediate early gene expression may precede obvious nuclear morphological changes and tissue functional decline, providing potential clues for early disease detection and therapeutic-window assessment. Testing these predictions will require integration of live-cell mechanical measurements, allele-specific editing, engineered tissue models, and single-cell and spatial multi-omics to establish quantitative causal relationships from mechanical input to nuclear output.

The multilevel functions of the LINC complex also create new translational opportunities for precise mechanical intervention. For states dominated by mechanical uncoupling and insufficient adaptive transcription, restoring specific SUN-KASH connections, stabilizing tissue-specific Nesprin isoforms or rebuilding normal perinuclear cytoskeletal organization may help restore physiological force transmission. Conversely, in LMNA-associated cardiomyopathy models, attenuation of pathological LINC coupling has also shown protective effects *in vivo*. Cardiomyocyte-targeted AAV9 delivery of a dominant-negative SUN1 miniprotein (DN-SUN1) disrupted endogenous SUN-KASH interactions and increased longevity by at least fivefold in Lmna-deficient mice, providing direct *in vivo* proof of concept for modulation of the LINC coupling interface (Chai et al., 2021). Further *in vivo* studies showed that cardiac Sun1 knockdown improved cardiac function and prolonged survival, whereas competitive expression of the Nesprin-1 KASH domain improved ejection fraction and reduced cardiac fibrosis in Lmna cardiomyopathy models, further supporting the SUN-KASH/LINC interface as an actionable intervention target (Tan et al., 2023). Beyond the LINC coupling interface, the nuclear lamina and nuclear load-bearing machinery may represent another level of intervention. In neonatal Lmna−/− mice, AAV-Lmna/DJ8-mediated re-expression of lamin A restored its proper nuclear-envelope localization, partially improved left-ventricular systolic function and prolonged survival, providing *in vivo* evidence for LMNA gene replacement as a complementary strategy at the nuclear load-bearing end of the LINC-lamina axis (Izawa et al., 2026). Existing mechanistic studies also suggest that enhancing nuclear lamina buffering, regulating chromatin mechanical state and correcting abnormal nucleocytoplasmic transport may provide complementary approaches to restore mechanical homeostasis at the nuclear load-bearing end and at the nuclear response end, although their therapeutic effects still need to be validated in disease-specific models (Elosegui-Artola et al., 2017; Jabre et al., 2025; Nava et al., 2020). At present, however, no LINC-targeted therapy has been established for clinical use, and the intervention strategies discussed above should be regarded as preclinical proof-of-principle requiring further validation in disease-specific and human-relevant models. Future therapeutic strategies should therefore not aim to universally enhance or inhibit the LINC complex. Instead, they should stratify and selectively modulate abnormal mechanical pathways according to genotype, affected tissue, dominant force-transmission branch and disease stage. The context-dependent mechanisms, tissue-specific disease outcomes and potential preclinical intervention framework are integrated in Figure 4.

**FIGURE 4:**
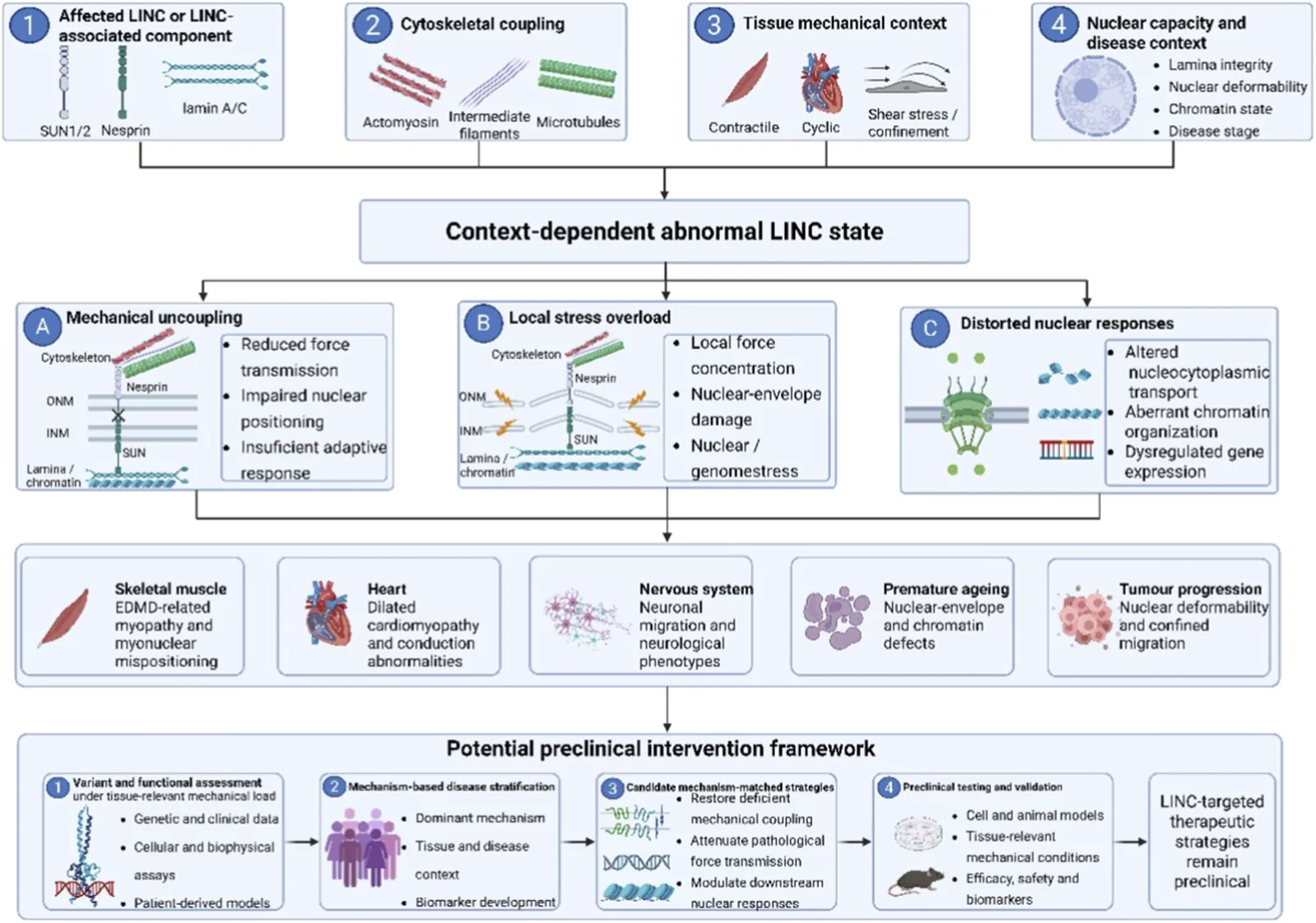
Context-dependent LINC dysfunction, tissue-specific disease outcomes and a potential preclinical intervention framework. The consequences of LINC dysfunction depend on the affected component, cytoskeletal coupling branch, tissue mechanical context and nuclear load-bearing capacity. Their interaction may produce mechanical uncoupling, local stress overload or distorted nuclear responses, contributing to muscle, cardiac, neurological, ageing-related and tumour phenotypes. Potential preclinical steps include tissue-relevant variant assessment, mechanism-based stratification, candidate mechanism-matched intervention and preclinical validation. LINC-targeted therapeutic strategies remain preclinical.

Overall, the LINC complex can be conceptually viewed as a dynamic mechanical matching interface between the cytoskeleton and the nucleus. It controls not only how mechanical force crosses the nuclear envelope, but also how physical input is converted into nuclear structural adaptation, chromatin remodelling and transcriptional responses. This framework integrates structural heterogeneity, mechanotransduction function, tissue selectivity and disease phenotype of the LINC complex into a single continuous mechanism, and provides a theoretical basis for distinguishing mechanical uncoupling, local stress overload and abnormal conversion of nuclear responses. With further development of *in situ* mechanical measurements, patient-derived tissue models and multiscale omics technologies, research on the LINC complex is expected to move beyond structural connection mechanisms toward disease stratification, identification of candidate mechanical biomarkers and development of precise intervention targets.
